# Expert clinician’s perspectives on environmental medicine and toxicant assessment in clinical practice

**DOI:** 10.1186/s12199-018-0709-0

**Published:** 2018-05-16

**Authors:** Nicole Bijlsma, Marc Maurice Cohen

**Affiliations:** RMIT, School of Health and Biomedical Sciences, PO Box 71, Bundoora, Victoria 3083 Australia

**Keywords:** Environmental medicine, Environmental health, Clinical practice, Exposure assessment, Environmental sensitivities, Toxicant assessment, Multiple chemical sensitivity, Exposure history, Toxicant biomarkers, Occupational and environmental medicine

## Abstract

**Background:**

Most clinicians feel ill-equipped to assess or educate patients about toxicant exposures, and it is unclear how expert environmental medicine clinicians assess these exposures or treat exposure-related conditions. We aimed to explore expert clinicians’ perspectives on their practice of environmental medicine to determine the populations and toxicants that receive the most attention, identify how they deal with toxicant exposures and identify the challenges they face and where they obtain their knowledge.

**Methods:**

A qualitative study involving semi-structured interviews with expert environmental clinicians in Australia and New Zealand was conducted. Interviews were recorded and transcribed, and themes were identified and collated until no new themes emerged.

**Results:**

Five dominant themes emerged from 16 interviews: (1) environmental medicine is a divided profession based on type of practice, patient cohort seen and attitudes towards nutrition and exposure sources; (2) clinical assessment of toxicant exposures is challenging; (3) the environmental exposure history is the most important clinical tool; (4) patients with environmental sensitivities are increasing, have unique phenotypes, are complex to treat and rarely regain full health; and (5) educational and clinical resources on environmental medicine are lacking.

**Conclusions:**

Environmental medicine is divided between integrative clinicians and occupational and environmental physicians based on their practice dynamics. All clinicians face challenges in assessing toxicant loads, and an exposure history is seen as the most useful tool. Standardised exposure assessment tools have the potential to significantly advance the clinical practice of environmental medicine and expand its reach across other clinical disciplines.

## Background

Epidemiologic studies, breakthroughs in biomarker research and large biomonitoring studies have raised awareness of the impact of environmental exposures and their relationship to chronic illnesses. Yet, whilst most clinicians acknowledge that environmental toxicants affect human health and are frequently asked about exposures by their patients, a lack of environmental health training and standardised exposure assessment tools leaves most clinicians feeling ill-equipped to assess or educate patients about toxicant exposures and their consequences [1–3].

Expert environmental medicine (EM) physicians not only require skills in clinical medicine, they also require specialised knowledge about the impact of different toxicants on human health, exposure sources and dose estimation, the factors that influence inter-individual variation to toxicant exposures, interpretation of laboratory tests, measures to minimise toxicant exposures and interventions that treat different sequelae. EM therefore requires the integration of knowledge from diverse fields and the skills to apply this knowledge in a wide variety of circumstances, yet this skill set is poorly defined and it is unclear how experts apply their knowledge to the needs of their patients.

Whilst there are text books on occupational and environmental medicine [4, 5], to date, there has been very little literature on the clinical practice of EM and little qualitative research on environmental clinicians’ perspectives. To address this, we undertook a qualitative study of environmental physicians with the aim to determine the nature of EM practice and identify how expert EM clinicians deal with environmental toxicant exposures. We further aimed to determine where clinicians obtained their knowledge and skills, the populations and toxicants that receive the most attention and the challenges they face in order to inform the development of resources and tools that can be used by all clinicians.

## Methods

A qualitative study was performed that involved a series of one-on-one, in-depth, semi-structured interviews with clinicians with an undergraduate degree in medicine who were identified as experts in the field of EM.

### Participant selection

Potential participants were identified by contacting doctors (via phone and/or email) known to be prominent in the field of EM through their speaking at conferences, lecturing in postgraduate courses and membership to either the *Australasian College of Nutritional and EM* (ACNEM) or the *Australasian Faculty of Occupational and EM* (AFOEM), which are the only two Australian medical organisations with ‘EM’ in their title. Some of the participants were known to the researchers prior to commencement of the study through mutual participation in conferences. This was followed by a snowball recruitment campaign whereby participants were asked to identify further experts. In addition to contacting prominent individuals, doctors listed on the ACNEM and AFOEM websites were emailed and invited to participate. A follow-up phone call was made to those who responded to explain the nature of the research and invite their participation. All prospective participants were emailed a *Project Information Statement* and contacted to make an interview appointment. Clinicians were not compensated for their time.

### Survey design

A series of open-ended questions was developed by the authors to determine:

- The nature of their EM practice
  - ○ The type of diseases they treated
  - ○ How much time they spent with patients
  - ○ The cost of consultation and tests
  - ○ The populations and toxicants they focus on
  - ○ Their greatest successes and challenges
- How they assess environmental exposures
  - ○ The most effective tools they use
  - ○ The type of tests they undertake and how they interpret them
- Where EM clinicians obtained their knowledge
  - ○ The institutions and associations to which they belonged
  - ○ The journals, websites and books used
  - ○ The educational and clinical resources used

### Interview process

Interviews were conducted via Skype audio by the first-named author during which the clinicians provided informed consent. There were no other participants present and no follow-up interviews. General questions (tell me about your practice) initiated the conversation, and open-ended questions were used to give the participant the freedom to explain their responses and identify new themes. The interviews were recorded and transcribed, and a preliminary analysis of the initial interviews suggested new questions for the next interviewee as specific themes became apparent. Recruitment of clinicians continued until saturation had been reached (no new themes emerged).

### Analysis

NVivo 11.3 software program was used to document specific themes within each interview, and these were analysed to identify dominant themes common across the entire cohort. The results were reported according to published guidelines for reporting qualitative research [6].

## Results

A total of 16 clinicians participated in the study: 11 integrative medical practitioners (six males and five females) and five male occupational and environmental physicians. Thirteen clinicians were based in Australia, and three integrative medical practitioners were based in New Zealand. The average length of EM practice was 20 years.

The themes that emerged from the data analysis were:

1. EM is a divided profession
2. Clinical assessment of toxicant exposures is challenging
3. The environmental exposure history is the most important clinical tool
4. Patients with environmental sensitivities are increasing, have unique phenotypes, are complex to treat and rarely regain full health
5. Educational and clinical resources on EM are lacking

### Theme 1: EM is a divided profession

The strongest theme is that environmental medicine clinicians can be classified into two distinct groups: integrative medical practitioners (IPs) and occupational and environmental physicians (OEPs) based on the nature of their employment (patient versus corporate-centered), the patient populations dealt with, the type of diseases they see, the type of toxicants they were concerned about and their views on the role of nutrition and genetics in toxicant exposures. Comments from clinicians that illustrate the differences between IPs and OEPs are presented in Table 1.

**Table 1:** Environmental medicine is a divided profession

Area of difference	Clinician	Quotes
Nature of employment	OEPs	I’ve never worked in the private clinical area as a fee-charging professional. A lot of our fellows do medical-legal work and don’t do much work in the environment space. We do more work-related than actual environmental toxins. We reviewed the use of copper beryllium in the aviation industry. Most court cases I do, are usually exposures to substances that are used in workplaces. I do two days with private cases, referred to me by general practitioners which includes worker’s compensation in motor vehicle accidents assessments. It has an emphasis on musculoskeletal medicine, but from time to time, I will see some cases with Multiple Chemical Sensitivity. There are very few people out there who can find us and see us as environment medicine specialists. I don’t get many referrals, may be one a month and people somehow they find their way to me, maybe even less. You have to walk this very fine line where you have to tell the management to do things and you have to tell the workers to wear their hearing plugs and masks. So you’re not very popular with anybody. Most of us are more closely aligned with the management side. Our job is to really protect the workers, but I think a lot of us have moved too far to the right. I think I was a bit too critical at one stage and so I lost my job. You’ve got to understand that the older guys have been welded to these management of the coal and gas. That’s where their money is coming from all their life, and they’re not going to start turning on their companies just yet and start advising them to close down.
	IPs	The patients that I see have seen seven or eight different doctors and have been examined to death and nothing has come out of that. The basis of my practice is chronically ill people, who do not fit into a clear medical diagnostic category. I see more kids with, rather than an obvious diagnosis, just a splattering of all sorts of things not quite right.
Type of diseases seen	OEPs	Mainly chronic neck pains and chronic back pains from a work injury. So it’s much more musculoskeletal than environmental unfortunately. A lot of our fellows are heavily involved in musculoskeletal injuries, noise, slips, trips and falls. A lot of our doctors will be doing noise exposure history. They’ll (the worker) be deaf from being at work and just take down the history and do the audiometry and send in for their compensation. The work I do with mining in New South Wales is a couple of cases of pneumoconiosis in coal miners.
	IPs	The basis of my practice is chronically ill people, who do not fit into a clear medical diagnostic category… [they] suffer [from] persistent inflammation, immunological dysregulation and neurological responses which are hyper-responses. Chronic and complicated, ill-defined conditions like Chronic Fatigue and tiredness, chronic allergy intolerances and autoimmune dysfunction. A mixture of the really kind of severe multiple chemical sensitivities, which I see a number of, where they come in, with a proper mask on the whole time, because they didn’t get through the waiting room to my office without being affected, to the much milder versions of that. Kids who are affected, but not full on the spectrum… we’re just seeing a bigger cohort of those more subtly affected. Multiple sclerosis, Motor Neuron Disease, Parkinson’s disease, those would probably be the ones I see most, and neurological dysfunctions that don’t necessarily get a name because they are somewhat atypical. I see quite a few kids that live in a house with mold. If a child presents with explosive behavior, or problems with focus, attention and judgement, the first thing I do is make sure it’s not a reaction to chemicals.
Toxicants of concern	OEPs	The environment means different things to different people. What we’re talking about is a workplace environment. You can have significant environmental exposures, but by and large, it’s the ones we know about, you know, the lead, the mercury, the radioactive stuff. Most of these are heavy metals. I’m doing a coal (medico-legal) case at the moment for coal dust. I’m looking at people who were aircraft fitters and maintainers who in the course of their duly entered fuel tanks and were exposed to the various combinations of military aviation turbine fuel. Exposures to substances that are used in workplaces like caustic or acid substances… also occupational exposures that are carcinogenic substances. Solvents and pesticides are still one of the issues that comes up, but much less than it did in the 1990’s because of a shift to ‘softer’ chemicals, implementation of OHS regulations and changes in the method of application (moon buggies rather than aerial application). Hormone disrupting chemicals are not a consideration in our industry. We don’t deal with the long-term… because this is what normal life is. None of these very, molecular or unseen chemical injuries are being monitored, because that’s still really research. If you’re talking about the millions of chemicals that are produced, not many actually have good known studies. Gold standard studies are rare as hen’s teeth on what these things actually do to people. I have been struggling with things like electromagnetic radiation and so on. And those arguments have gone on for thirty years or more, without any definitive answers really.
	IPs	It’s the very ubiquitous ones that people are routinely eating and drinking, without thinking much about it, just because of it being in the water supply and food. I think the biggest issue with environmental toxic load, is the amount of processed food that we eat, including foods stripped of nutrition, foods stripped of fibre, foods laced with food chemicals, foods dowsed in pesticides. Foods using abnormal hydrogenated toxic oils. I think that is actually our biggest danger. Most people expect toxins are outdoors, but most of the toxins are indoors. Mold is very common, chemicals and some degree of heavy metal exposures are very common as well. So, it seems to be that the mix of it all, the toxic synergy creates a bad result, rather than any single substance on its own. I see quite a few kids that live in a house with mold… you get them out of that house and they get better. There was a period in the early 1980’s, where the organochlorine pesticides heptachlor and chlordane were required by law to be used under the slabs of homes as a termite treatment, and lots and lots of people got sick. If I’ve got someone with really intractable hormonal issues, breast or prostate cancer, I’d be looking for exposure to plastics and pesticides and some of those xenoestrogens. Problems with metal allergy, not just metal toxicity. So, if you take inorganic mercury from dental amalgams it can be a mitochondrial poison, it can inhibit a vast array of enzymes. Plenty of times I’ve been looking for external toxins, there’s nothing around and then the whole process unravels and it turns out that they have a fear of spiders and they’ve had the house completely sprayed (with pesticides) in every single room. Children eat a lot of fish, particularly Asian children, so much higher rates of mercury toxicity. All of those children (with neurobehavioral disorders) will have a proportion of their problem due to foods and chemicals. If you figure out that this child is reacting to a food, or a chemical and you remove that food or chemical, then, on review, you can untick all those boxes … how I regard those children, is not as having autism or ADHD, that is, really, as having food and chemical sensitivities, that express themselves with those conditions. I see much more of the indoor air pollutants and much less of the agricultural chemicals.
Role of nutrition in toxicant exposures	OEPs	There’s a fringe thing called nutritional medicine... it’s not really recognised. There is this serious discrepancy here, between Occupational and EM and this fringe group who call themselves EM practitioners. I don’t spend my time talking about nutritional enhancement of their condition, whatever that is. With people that don’t have a recognised problem such as an allergy, nutrition is unlikely to play a role. I stick to the big things like fish and mercury, pesticides in fruit and lead exposures if they haven’t washed their hands this may potentially contaminate the food.
	IPs	Nutrition is your backbone in biochemistry; if you’ve got good nutrition, you’ve got a level of resilience against environmental insults, if you’ve got poor nutrition, you have less resilience. When you go from healthy food to processed food, you are doing two things: you’re decreasing the nutrients and at the same time increasing the toxins, and this changes the whole balance of survival. If a person is low in calcium and iron, they have a greater affinity to absorb lead. Likewise, if a person is deficient in Vitamin C and selenium, they have a greater affinity for absorbing mercury. If you eat a healthy diet, high in antioxidants, you’re more likely to be able to detoxify and get rid of those toxins… if you eat an organic diet, you reduce your pesticide load; when you eat fish, you are more likely to be exposed to heavy metals and other pollutants. So nutrition has a huge amount to do with that. If the body has optimum nutrition, then the toxic load is less likely to be problematic. Certain toxins bind up our enzymatic pathways and create nutritional deficiencies. So nutrition is really important for protecting you against toxic exposure. Zinc and manganese are very important for upregulating metallothionein, and in protecting you against heavy metal exposure. If the child is iron deficient, they have increased absorption of lead and often have pica symptoms, of eating the dirt. Food is probably the first one because I know how toxic gluten is… it is associated with inflammation. The biggest issue with environmental toxic load, is the amount of processed food that we eat, including foods stripped of nutrition and fibre, foods laced with food chemicals, foods dowsed in pesticides. Foods using abnormal hydrogenated toxic oils. I think that is actually our biggest danger.
Attitude towards genetic testing	OEPs	There’s a huge reluctance in occupational and EM to do genetic testing because of the implications, discriminating against people on the basis of genetic predisposition to problems. Are we in the position to actually do the testing and make those decisions? No, that’s very contentious.
	IPs	People with the HLA DRB-1 and the HLA-DQ test do put them into a category that makes perfect sense about why the person reacted (to biotoxins in a water-damaged building). I really do think that the idea of checking the genetics and susceptibility will be a big thing, once we understand why those particular chromosomal changes predispose a person towards more toxic injury than others. The genes that I usually look out for would be the methylation genes, the glutathione-related genes, the Phase I and Phase II hepatic detox pathway genes, the acetylation genes of glucuronidation pathways, the Metallothionein gene and PON-1 (Paraoxonase-1). I have a sense that neurological sensitivity and methylation disorders and hypersensitivity to toxins, are different aspects of the very same thing, of the very heightened response of the central nervous system to particular inputs.

The nature of employment between the two groups was quite distinct. The IPs were general medical practitioners (*n* = 9) and paediatricians (*n* = 2) who worked in private practice and averaged five or more patients per day. IPs were directly employed by the patient, and the average cost of an initial consultation was $AUS 421 (this varied from $280 to $630) and the average subsequent consultation cost was $AUS 269 (this varied from $150 to $360). In contrast, the OEPs received a salary from an employer as company physicians in the aviation, military, mining, oil, gas or manufacturing industries to assess and monitor workers’ health or as advisors in government departments or emergency physicians in hospitals or an agreed fee to conduct medico-legal work to assess causation or disability for insurance companies or workers’ compensation or motor vehicle accident claims.

The OEPs primarily deal with adult men with musculoskeletal disorders and diseases arising from occupational exposures to heavy metals, asbestos, coal dust, beryllium, pesticides or solvents such as benzene, diesel and isocyanates. In contrast, the IPs saw patients with chronic, complicated ill-defined conditions involving multiple systems characterised by long-term fatigue (chronic fatigue syndrome, multiple chemical sensitivity and fibromyalgia), allergy intolerances, digestive disorders and chronic autoimmune, metabolic and/or neurological conditions along with children with learning, developmental and behavioural issues and recurrent infections.

Whilst all clinicians agreed toxicants are harmful, there was a stark difference in the type of toxicants they were concerned about. OEPs were primarily concerned about acute and chronic exposures arising from the workplace or hobbies where linear dose-response relationships are well described. In contrast, the IPs were concerned about long-term exposure to low-level toxicants in food, the workplace and the home environment and their combined effects. The two groups also differed in their perspectives on nutrition. OEPs considered nutrition as fringe medicine, not related to toxicant exposure except for special cases such as mercury in fish, pesticides in fruit and contamination of food with lead dust. In contrast, all the IPs highlighted food as the most important source of toxicant exposure and as a treatment to build resilience.

Whilst all clinicians acknowledged that genetics was important, most of them did not do genetic testing. None of the OEPs conducted genetic testing due to the costs involved, clinical uncertainty, lack of knowledge on gene variants and the ethics involved in discriminating against people. In contrast, four of the IPs conducted genetic testing in a minority (5%) of their patients with chronic, idiopathic environmental sensitivities.

### Theme 2: Clinical assessment of toxicant exposures is challenging

Comments that indicate the challenges in assessing toxicant exposures are presented in Table 2*.* IPs noted that EM requires long consultation times that limits the number of patients that can be seen, leading to long waiting lists and high costs, which are compounded by the costs of specialised laboratory tests not being covered by third-party reimbursement. Toxicant and other laboratory tests were also noted to be unreliable, unavailable and lacking standard clinical approaches.

**Table 2:** Clinical assessment of toxicant exposures is challenging

Key challenges	Clinician	Quotes
Lack of clinical guidelines	IP	It would be great to have a standardized data-collection tool for environmental exposure history. It would make an enormous difference to outcomes. I’ve got a patient today with high bisphenol and phthalate levels. What do you do about it, besides stopping the exposure? So then, the question is, when they’ve got all these things and they’ve stopped the exposure and they are still very sick people, how do you go about dealing with that?
Limitations of laboratory testing	OEP	The frustrating thing with EM is actually trying to find the tests which can actually show that what they (the patient) have got is real. You would counsel (the patient) against over testing and wasting public money. Most of them you don’t have tests for. Testing is really difficult. I have a very limited array of tests. If you’re trying to test for things like benzene exposures and stuff, you really have to catch that at the time they’re exposed because very quickly you don’t get many metabolites left in the system from solvent exposures. It’s far too expensive for my patients to do that. I use blood for heavy metals and obviously that only picks up the products that are long-term exposures that bioaccumulate like lead, cadmium, and nickel. I just don’t feel like I have the tools to do a better job.
	IP	There’s a lot of rubbish pathology that goes on, where we don’t have good standards, where we accept almost any kind of result as proof of poison and abnormalities and we don’t have good, validated ways of understanding how to measure the toxins. So we don’t have good surrogate markers, because no one can agree on what a marker of a toxic exposure does. The whole question of the testing… it’s really a mine-field that’s very hard to get your head around. Standard medical labs do not measure anything that are specific to environmental toxins or exposures. In my area of medicine (CFS/MCS), physical examination is terribly disappointing. It doesn’t really show much at all and that’s part of why people get ignored in this area. They can have neurological and immunological impacts and the physical examination looks and feels just the same as any other person. Although we wanted to believe that the Australian labs were doing it properly, clearly they weren’t, when they had five-fold differences in one-split sample which they thought were different patients. I used to use porphyrin tests, but I found that the results were so inconsistent, that I just stopped. I don’t test for chemicals. I just do the functional liver detoxification profile and get their livers working properly to get rid of the chemicals.
Difficulty in establishing cause and effect	OEP	In case of punitive or suspected cause, until there’s sufficient scientific evidence supporting, relating the possible cause and the effect... that’s where dose-response relationships are important, whether there’s a plausible biological mechanism that can explain the mechanism from the exposure. It’s important not to create alarm. It’s hard to quantify these health risks. I think what happens in a lot of companies is you do what you’re regulated to do, and then you just report what you can. There’s a lot of uncertainty in everything we do. We simply don’t have enough data to make strong conclusions unfortunately. You need to stick with science as much as possible. But there are a lot of areas where the science isn’t that great.

The OEPs reported their most significant challenge with toxicant testing was the difficulty in establishing cause and effect. In contrast, many IPs undertook tests with uncertain clinical meaning such as hair mineral analysis, digestive stool analysis, organic acids test, provoked challenge urine test, food sensitivity tests, lymphocyte sensitivity test, liver detoxification profiles, tests for specialised inflammatory markers associated with biotoxin exposure and tests to detect persistent organic pollutants or tick-borne diseases.

### Theme 3: The environmental exposure history is the most important clinical tool

It was agreed by all clinicians that the environmental exposure history is by far the most important clinical tool for assessing toxicant exposures. The average time clinicians spent taking an exposure history was 90 min although this varied from 1 to 3 h. Some clinicians used formal questionnaires, whilst others relied on patients’ responses to guide their questioning. Clinicians’ comments on the relevance and characteristics of an environmental exposure history are presented in Table 3.

**Table 3:** The environmental exposure history is the most important clinical tool

Features	Clinician	Quotes
Importance of an environmental history	OEP	It’s quite a long, drawn out question and answer process that follows no specific format, because then I go along and choose the questions I want to ask, to determine what is their alleged level of exposure and what has been the responses by their body to that exposure, and what measurements they’ve had.
	IP	History, history, history is the most important tool to use. I tell the patient, it’s all in the history. Let’s spend some time getting it down and also that we’re not going to get this right in, like, the first appointment, it might take several appointments. Because we couldn’t rely on the Australian laboratories, we stopped testing and went entirely on history. It’s important to ask a very detailed history: occupational, hobbies, recreational, nutritional, environmental exposures, even down to things like chemical use in the house, indoor air pollution, external air pollution etc. It’s becoming less and less value to do toxin checking and much more valuable to say is your history one of exposure to toxins and if so, what is the generalised approach that we could do, to safely protect and unload you from whatever the likely historical toxins are. The first thing is to have an awareness of the possibility (of toxicant exposure). The second is to ask a good history. And then trying to find out what tests could be done and what treatments could be used or who to refer to.
Challenges of long consultations	OEP	In 90 minutes you can actually really get to understand a person and their risk of exposures and whether in fact this might be something that is related to occupational or environmental exposures. We’re taught to ‘Take a good history’. I would challenge most doctors now, they don’t. They’ve got four minutes to see a patient, they’re supposed to see forty a day, get them in, get them out. If there’s something more complex, they say Oh, I’ll tell you what to do, let’s get a blood test done here, or Let’s see how you go, take these two pills and I’ll see you in two weeks, if you’re not better. And then they hope in two weeks, that they’re going to find more time, but they don’t.
	IP	90 minutes allows me to give an hour to the history taking portion and half an hour for examination. That’s really what’s needed to conduct a proper environmental history. With the existing system, the history-taking part has to be done in about two minutes, then you have to get the blood pressure on and the script, or the investigation printed within five minutes and then your ‘closing statement’ is six, or seven minutes. There’s no way you’re going to pick up anything deeper in that time… and it’s just enough history to work out which medication or investigation may be. So, that is a big problem. The system isn’t really set up for doctors’ seeing patients for a long period of time. And if you claim for a long consultation, they complain.
Occupational history	OEP	You need to go through what is this person exposed to through the whole course of their working life. I would tease it out, detail by detail, according to the history given, whether it was volatile organic solvents, whether it was dust, whether it was asbestos, whether it was diesel fumes. So it would become an individualized, personal question-and-answer, to get a measure of what the exposure has been, in both the long-term and in the short-term, resulting in the symptoms as expressed by the individual. You ask people what they are exposed to, then you look at specific heavy metals or chemicals in the workplace. If somebody was making or refurbishing old thermometers, then test for mercury. If they’re working on bearings or grinding in a machine workshop, you’d probably do lead and cadmium. If they’re doing spray painting for corrosion control on a metal aircraft, then you do chromium. So you tailor it to the environment. If you were a painter, preparing, or getting rid of lead paint in old houses, first of all you scrape the old paint and then you burn it. And if you don’t do it properly, you could be exposed to significant lead levels from the old paint.
	IP	If they have listed a job involving the use of chemicals, farming, soldering, or various things like that, I’ll specifically ask what personal protection they use. I’ve noticed that some of the parents with autistic children are often very intelligent people in high end academic jobs, but not in great locations like an oil rig and things like that. People working around swimming pools and golf course green-keepers, were getting sick with the same illness’ that the farmers in the Central Coast were getting years ago.
Place history	OEP	Mount Isa mines in Queensland and Port Pirie in South Australia they’ve shown quite significant spread (of lead dust), many kilometers from the stacks and waste dumps.
	IP	Where they’ve lived as children, renovations of houses, all those kind of things don’t actually come out unless you ask that. You need to investigate the house for lead paint, or eating antique furniture, being bathed in an old bath. Where were you born, where did you grow up, what were your early life experiences and exposures, or potential exposures, to toxins, what was your health like in childhood, early adulthood and adulthood? I get them to map out on Google maps where they live, go to school and where they work. I draw a sausage shape around their home, and go half a kilometre sideways and one kilometer each end-wise and find out what is the vicinity of golf courses, industrial areas, service stations, main roads, airports, farms, bowling greens, parks…? What are the prevailing summer and winter winds? although wind direction is not useful in hilly areas. And so, we basically just stare at the map on the places where they used to live and work, and where they currently do live and work. It’s a useful thing for identifying where very sensitive people should buy houses or rent. In relation to the Chronic Fatigue Syndrome cluster around Botany Bay, we identified hot-spots for hexachlorobenzene at Botany Bay and dioxin and PCB exposure around Homebush Bay. What school did you go to, where was it? Lots of the toxicological assaults come from the schools, which can be situated on hills, or beside main roads and kids get plenty of exposure to cleaning agents and traffic fumes, pesticides and just about everything there. And then, half the schools seem to have the old, unflued gas heating systems through all of winter… volatile organic chemical exposure and respiratory irritants are high.
Dietary history	IPs	So tell me a bit about the chemical reaction you are concerned about. When was it, how long was it, how long after the exposure, the duration of the effects. What did those effects, i.e. was it gas, or the neurological. you know, childhood, behavioral. And then I just try and map it out. Then you go on to the next one. What was the next environmental reaction which your child had. And then you just slowly build up a picture of the person, and then I go through the artificial chemicals and colors, additives and preservatives as well as the natural colors, flavors, preservatives, like salicylates. And then, I go through the family history as well, that’s very important. I look for genetic predisposition and for chemical sensitivity. If they are salicylate-sensitive, they’ll usually have a reaction to other chemicals, to artificial colors, flavors, additives. And the typical child, you know, the blond-haired, blue-eyed freckly, or red-head,...If you see someone like that and they’re of Scottish origin and they’ve got behavioral problems, that’s the first thing I go to.

The exposure history encompassed a variety of questions about the patient’s occupational, dietary, dental, drug, lifestyle, hobbies and place history. Whilst there were many similarities in the type of questions asked, the OEPs spent more time obtaining a detailed occupational history, whilst the IPs spent time obtaining a comprehensive dietary history. Place history was considered to be important by both groups; however, whilst the OEPs focused on occupational exposures from workplaces, IPs focused more on exposures to traffic-related air pollutants, pesticides and other toxicants from commuting, workplaces and homes.

### Theme 4: Patients with environmental sensitivities are increasing, have unique phenotypes, are complex to treat and rarely regain full health

Where IPs treated patients with chronic idiopathic environmental sensitivities, OEPs were more likely to attribute symptoms to psychological factors on the basis that linear dose-response relationships could not explain their symptoms. Consequently, information about patients with environmental sensitivities was mostly derived from the IPs, and their comments are presented in Table 4.

**Table 4:** Patients with environmental sensitivities have unique phenotypes, are complex to treat, rarely regain full health and are becoming more prevalent

Key challenges	Clinician	Quotes
Attitude towards patients with environmental sensitivities	OEPs	We don’t deal with the long-term because this is what normal life is. None of these very, molecular or unseen chemical injuries are being monitored, because that’s still really research. There often is a lot of worry and psychological overlay with all of these cases, as they’ve been hunting around for months to find out what’s wrong with them. You have to be a little bit careful in this area otherwise you can get a (bad) reputation. Dose-response relationships are important. Whether there’s a plausible biological mechanism that can explain the mechanism from the exposure... It’s important not to create alarm. I’ve seen a few multiple chemical sensitivity cases and you know most people write them off, but I tend to feel that there’s something going on there. There may be a psychological overlay; I don’t deny that, but there’s often a triggering event. There’s only a few of us that drifted more towards a better understanding of patients holistically.
	IPs	The canaries are the ones that are set up early on in life to have more difficulty dealing with the environment, because in terms of liver and cellular detox, they’re not that well-equipped for the environment. So in terms of the bell-shaped curve, they’re at one end. And then the rest of us are in the rest of the bell-shaped. And then there’s the bulldozer, these guys go through life and they smoke and drink and they spray everything and you have to run them over at ninety. Could it be overexposure therefore causation because the dosage was high enough, or could it be failure of elimination, because in the genetic diversity of the human race, some people are just crappy at clearing drugs, pills, potions and pesticides out of their system. As you go through life, many things you’ll get over. But other things are there as a toxic record, if you like. Toxic Load. So it’s kind of the building blocks. And you get to a point, where it takes just a small event, whether it’s a viral infection, or some other toxic event, that pushes them through their ability to compensate and then they can go off in various directions, whether it’s chronic fatigue, autoimmune disease, degenerative disease, cancer, they are just options thereafter.
Observations of patients with environmental sensitivities	IP	I didn’t think see those allergies ten years ago. The whole ADHD has been kind of like a tsunami in the making in recent times. The increased number of people with mold-related illness… I do see more of what I now appreciate biotoxin exposures, rather than all pesticides, poisons and other types of toxins. They’ve become a lot more difficult; more chronic illness, more environmental intolerances, more food intolerances and allergies, more chemical sensitivity… a rise of auto-immune diseases. The biggest thing that has changed is the degree of education. The patients are much more aware and now they are far more likely to seek advice and tend to come earlier. Thankfully patients are getting more informed about ideas and will often come in, rather than I’ve got something terrible happening and I’ve got no idea what it’s about, they will often be saying, I’ve got some terrible problems and I’m wondering about this, that and the other.
	OEP	Greater awareness of the population generally...I think GPs are becoming more aware of things like MCS and fibromyalgia… and for which patients were often rubbished thirty years ago.
Observations of phenotypes of patients with environmental sensitivities	OEP	The majority of people with Multiple Chemical Sensitivity have got some sort of an allergic, or highly reactive predisposition. It’s not so much how much toxin they’ve been exposed to; it’s the individual response to that which becomes important. One-fifth of the population who are already predisposed, develop this neuropathic pain, for which they have become a hypervigilant responder. They will give you a history of childhood asthma, maybe long-standing hay fever, working out in the farm areas with their exposures there. A lot of these people have an allergic background, and I think that shows that they’re at risk to (environmental) sensitization.
	IP	These patients are extraordinarily sensory-sensitive in every way… they tend to be artistic, highly creative, able to read another person, very sensitive to another person’s emotions. When I go on their history, sometimes it’s he was a normal kid, but he was very, very sensitive to whatever things are around. The blond-haired, blue-eyed freckly, or red-haired child of Scottish or Irish descent whose got behavioral problems are much more prone to salicylate sensitivity. And usually, if they are salicylate-sensitive, they’ll usually have a reaction to other chemicals, to artificial colors, flavors and additives. People of Scottish-Irish descent are much more sensitive to gluten than other people. The Irish for celiac disease, the Chinese for lactose intolerance is quite common. These are the individuals who come in wearing white gloves and masks over their face. They’re all very, very detailed-minded, perfectionists in their views on life. Sensitivity to smells and sensitivity to sound, to all of the senses, out of a group has an extraordinary advantage: that you hear the tiger, you know the poison in the plum, you become, effectively, the early warning radar. Their hypersensitivity might be a bit over the top but it keeps them out of harm’s way at a much higher rate than others... this may explain why the cancer rate in my patients is almost zero.
Observations of genotypes of patients with environmental sensitivities	IP	Some people are just crappy at clearing drugs, pills, potions and pesticides out of their system. And those who have it remain for a long period of time, may have vulnerabilities. People with the HLA DRB-1 and the HLA-DQ test do put them into a category that makes perfect sense about why the person reacted (to biotoxins in a water-damaged building). I have a sense that neurological sensitivity and methylation disorders and hypersensitivity to toxins, are different aspects of the very same thing, of the very heightened response of the central nervous system to particular inputs. A reaction to chemicals implies they have some sort of genetic defect in their Phase II detoxification pathways.
Difficulties treating environmental sensitivities	IP	The more symptoms and systems involved and the more chronic the illness, the more challenging it is; especially people with multiple chemical sensitivity, because then they have difficulty tolerating the treatment as well. Complicated chronic fatigue together with chemical sensitivity and pain… and severe neuroimmune dysfunctions. Those are the hardest to treat. Very few people that I see get cured for Chronic Fatigue Syndrome … maybe 90% or more of the Chronic Fatigue Syndrome and chemically sensitive people, never go back to the level of health that you would expect from a fit and healthy person of their age but they adapt well and are able to go back to life and do things with the knowledge of their limitations. Autism is clearly the hardest of the neurodevelopmental problems, complex neurodevelopmental disorders. Older severely autistic are the most challenging and also the severely allergic, especially with anaphylactic type reactions. Epilepsy, that’s probably the most difficult one. I’ve got a patient today with high bisphenol and phthalate levels. What do you do about it, besides stopping the exposure? So then, the question is, when they’ve got all these things and they’ve stopped the exposure and they are still very sick people, how do you go about dealing with that?

Several IPs noticed a significant increase in the incidence and awareness of environmental sensitivities like allergies, chemical sensitivities, neurodevelopmental disorders in children and mould-related illnesses over the past 10 years and commented that these patients were more likely to have gene variants in methylation and detoxification enzymes or specific haplotypes (HLA DRB-1, HLA-DQ) that made them susceptible to mould toxins and gluten.

Patients with idiopathic, multi-morbid diseases including chronic fatigue syndrome, multiple chemical sensitivity and autism were said to be the most difficult to treat due to challenges in limiting their exposures to everyday toxicants and poor tolerance to treatment, resulting in few of these complex patients regaining full health.

### Theme 5: Educational and clinical resources on EM are lacking

Clinicians’ comments on the lack of resources available on EM and the limitations in their training are listed in Table 5. Clinicians reported acquiring their knowledge on environmental toxicants over many years through many different sources which included formal postgraduate qualifications, online journals and websites, conferences and workshops, government organisations and institutes, collaboration with peers, discussions with patients and searches on PubMed and Google Scholar. Training also varied considerably across clinicians with no single resource or training program being recognised as comprehensive or an industry standard. All interviewed clinicians had an undergraduate medical degree and 12 had postgraduate qualifications, yet despite this training, it was acknowledged that EM is still an emerging field. Online journals and databases were seen as an important resource for most clinicians with Google Scholar, SNPedia and PubMed listed as the most useful. Local and international conferences conducted by the following associations or institutes were cited as a useful source of information: Australasian Faculty of Occupational and EM, Australasian College of Nutritional and EM, International Board of Clinical Metal Toxicology (The Netherlands), Australian College of Medical Nutrition, American Academy of EM (USA), Autism Research Institute (USA), Mindd Foundation (Australia), Clinical Education (UK) and the Institute of Functional Medicine (USA).

**Table 5:** Educational and clinical resources in environmental medicine are lacking

Resources	Clinician	Quotes
Education process	IPs	Difficult education process, everyone develops in their own way. There is no one single organization I think of as the best. And that’s why I go around to lots of different organizations and learn from all different sources. The resources just cover fragments and they specialise in one area. I find it very hard to find one organisation who does the whole picture. You have to put the fragments together.
AFOEM training	OEPs	My college has been very slow at developing the EM side but they’re working on it, but we’re not getting there fast. Recently-admitted fellows would not have very much training in chemical exposures, even at workplaces. The newer fellows (OEPs) really don’t know where to submit samples to for analysis.
ACNEM training	IPs	Good introductory course. Limited in their scope. We need a lot more in-depth teaching. It is probably the most comprehensive program at the moment (in Australia), but compared to what you can learn from abroad, from the States, it’s not as comprehensive.
Journals	OEP	Many of the things that come up (as an expert witness in court) require me to do quite a bit of research with Dr. Google and the online journals.
	IP	I haven’t found any (journals) that are very useful. I usually do searches and just try and pick up general articles when I am researching a particular topic.
Textbooks	IPs	I’m not aware of a really good environmental health textbook. Is there one? I’ve got toxicology textbooks but they tend to be far more involved with acute toxicity, rather than chronic, often low-level toxicity… a lot of what I think we see, is to do with the latter.
Peers	IPs	80% of what I learn, comes from a colleague emailing me, or passing on a paper from some source and then I go and read it and move on from the references there. I went to the Australian Society of EM’s annual meeting (now disbanded). About fifty to sixty other doctors would gather and talk and get lectured to and then go out and try to apply that elsewhere. So, the education was primarily through that group.
Patients	OEPs	Now I just learn from each case that I see. I still have a long way to go. There aren’t a lot of experts in this area. I teach general practitioners to listen much more to the patient, rather than get into your standard position of physical medicine.
	IP	I have my little army of a few thousand chemically sensitive, chemically poisoned, affected people. And I’m forever getting notices and emails like: Did you see that? Here’s the biomarkers of chemical sensitivity, Here’s what organochlorines do, There’s the glyphosate paper...

The websites and books mentioned by clinicians as useful resources in EM are listed in Tables 6 and 7, respectively. The OEPs focused on websites and textbooks relevant to occupational medicine and toxicology, whilst the IPs mentioned websites and textbooks dedicated to functional medicine and specific environmental hazards like mould and chemicals in consumer products. Peers, colleagues and patients were often mentioned as an important resource for information on EM. Numerous researchers and authors were mentioned as important sources of information along with government departments, laboratories and tertiary institutions.

**Table 6:** Websites used as a resource by clinicians on EM

Organisation	Website URL	Number of mentions
Environmental Working Group (USA) and their database Skin Deep	http://www.ewg.org/ http://www.ewg.org/skindeep/	× 3 (IP)
Surviving Mold (USA)	https://www.survivingmold.com/	× 3 (IP)
Autism Research Institute (USA)	https://www.autism.com/	× 2 (IP)
Environmental Health News Above the Fold (daily news feeds) (USA)	http://www.ehn.org/	× 1 (IP)
US Environmental Protection Agency	https://www.epa.gov/	× 1 (OEP) × 1 (IP)
Agency for Toxic Substances and Disease Registry (USA)	https://www.atsdr.cdc.gov /	× 1 (OEP)
REACH (Registration, Evaluation, Authorisation and restriction of Chemicals) (Europe)	http://ec.europa.eu/environment/chemicals/reach/reach_en.htm	× 1 (OEP)
Harvard (T.H. Chan) School of Public Health	https://www.hsph.harvard.edu/	× 1 (Paed)
US National Library of Medicine	https://www.nlm.nih.gov/	× 1 (OEP)
UK Health and Safety Executive	http://www.hse.gov.uk/	× 1 (OEP)
International Program on Chemical Safety. Environmental Health Criteria Monographs.	http://www.inchem.org/pages/ehc	× 1 (OEP)
International Labor Organisation	http://www.ilo.org/global/lang%2D-en/index.htm	× 1 (OEP)
Public Health England (UK)	https://www.gov.uk/government/organisations/public-health-england	× 1 (OEP
EnHealth (Australia)	http://www.eh.org.au/resources/knowledge-centre/enhealth-national-documents	× 1 (OEP)
Safe Work Australia	https://www.safeworkaustralia.gov.au/	× 1 (OEP)
National Industrial Chemicals, Notification and Assessment Scheme (Australia)	https://www.nicnas.gov.au/	× 1 (OEP)
US National Institute of Occupational Safety and Health (USA)	https://www.cdc.gov/niosh/about/default.html	× 1 (OEP)
Occupational Safety and Health Administration (USA)	https://www.osha.gov/	× 1 (OEP)
Friends of the Earth	http://www.foe.org/	× 1 (IP)

**Table 7:** Books on EM

Title	Author	Year	Number of mentions
Healthy Home Healthy Family	Nicole Bijlsma	2018	× 3 (IPs)
Chemical sensitivity. Volume 4.	William Rea	1998	× 2 (IPs)
Surviving mold	Ritchie Shoemaker	2010	× 2 (IPs)
Environmental disasters. A chronicle of individual, industrial and governmental carelessness.	Lee Davis	1998	× 1 (OEP)
Occupational medicine	Carl Zenz	1994	× 1 (OEP)
Four volume encyclopedia of occupational health and safety.	International Labor Organisation (ILO)		× 1 (OEP)
Hunter’s diseases of occupations (10th ed)	Peter Baxter and Tar-Ching Aw	2010	× 1 (OEP
Toxic metals and antidotes. The chelation therapy handbook. (2nd ed)	E. Blaurock Busch. MTM Publishing.	2012	× 1 (IP)
Clinical metal toxicology (12th ed)	Prof Peter van der Schaar	2015	× 1 (IP)
Casarett and Doull’s Toxicology. The basic science of poisons. (8th ed).	Curtis Klaassen		× 1 (IP)
Textbook of functional medicine	The Institute for Functional Medicine	2010	× 1 (IP)
Textbook of natural medicine (4th ed)	Joseph Pizzorno and Michael Murray	2012	× 1 (IP)
Detox or die	Sherry Roger	2002	× 1 (IP)

## Discussion

### EM is a divided profession

It appears that EM is divided between bottom-up patient-based approaches and top-down population-based approaches to medicine. This division is based on training and practice settings, which distinguish between specialists in integrative medicine (the IPs) and occupational and EM (the OEPs). These groups differ in the type of patients and the diseases they see, the type of toxicants they are concerned about, differences of opinion on the role of nutrition, and the type of pathology tests used.

The difference between the two groups may arise from their different education and practice dynamics. All of the IPs had studied nutritional medicine and were working from a bottom-up approach to assess and treat multi-morbid disease in the absence of clear evidence. These doctors were seeing susceptible individuals with chronic responses to low-dose exposures including women with idiopathic environmental sensitivities, patients with neurodegenerative disorders and children with neurodevelopmental disorders. In contrast, the OEPS all had formal training in occupational medicine and were employed by industry and insurance companies on a top-down approach with an emphasis on musculoskeletal disorders and occupational exposures to noise, asbestos, toxic metals, coal dust and various solvents in mostly adult male workers. These doctors considered linear dose-response relationships as the gold standard in establishing adverse health effects and were sceptical of patients with chemical sensitivities and ‘fringe’ doctors who use nutritional approaches and laboratory testing with uncertain clinical meaning.

### Clinical assessment of toxicant exposures is challenging, and the exposure history is the most important clinical tool

Whilst these differences give an appearance of a divided profession, there were strong similarities across all clinicians including agreement that an exposure history is the most important clinical tool, that EM is an emerging science with few established experts and that chemical risk assessment is challenging in the absence of standardised data-collection tools or guidelines for toxicant testing. Thus, clinicians questioned the value of tests with uncertain accuracy and clinical significance and favoured taking an exposure history over toxicant testing despite the time this requires. Whilst the elements of an exposure history varied amongst clinicians, they generally included questions about the patient’s past and current occupation, hobbies, lifestyle factors, diet, dental record and drug history. Several IPs stressed the importance of place history and the patient’s working, school and living environments. Despite its importance to clinicians and environmental epidemiologists [7], most clinicians were not formally taught to take an exposure history, and this is consistent with a report that only 20% of US paediatricians received training in environmental history taking [8].

### Patients with environmental sensitivities are increasing, have unique phenotypes, are complex to treat and rarely regain full health

Clinicians acknowledged that patients with environmental sensitivities often have unique phenotypes and that patients presenting with allergies (food and aeroallergens), neurodevelopment disorders and mould-related disorders appear to be increasing. This is consistent with evidence of an increase in the prevalence of allergic diseases [9–13], learning and behavioural disorders [14, 15] and mould-related disorders [16–21]. Clinicians also observed that these patients are more likely to seek help earlier on, are more informed about toxicants and are difficult to diagnose, complex to treat, and rarely regain full health. These observations are supported by a growing body of evidence on the complexities in diagnosing and treating patients with environmental intolerances such as chronic fatigue syndrome, multiple chemical sensitivity and systemic exertion intolerance disease, sensitivity-related illness, idiopathic environmental intolerances, fibromyalgia, electromagnetic hypersensitivity and sick building syndrome [22–27].

### Educational and clinical resources on EM are lacking

Despite the interviewed doctors all having extensive training in fields such as clinical medicine, public health, epidemiology, nutrition and occupational medicine, many felt inadequate to call themselves ‘experts’ or ‘environmental physicians’. This stems from their realisation that toxicant exposure assessment requires knowledge of highly complex clinical domains from genetics, nutrition, geomedicine, microbiomics and exposomics, which are not widely taught or integrated into clinical practice. Furthermore, environmental practitioners must diagnose and provide medical and nonmedical management for environmental diseases, translate new research results into practice and make complex causal inferences [28].

The complexities of EM are compounded by the lack of widely established educational resources with clinicians being unable to identify any single resource or training program that provides the knowledge they feel they need to practice EM. Consequently, clinicians were left to navigate their own path to acquire EM knowledge using a collage of sources including journals, books, websites, conferences, webinars and discussions with peers and patients. Remarkably, only one medical journal was mentioned as a useful resource, which highlights the lack of information on EM in general medical journals.

The perspectives of clinicians working at the coalface of EM highlights the many challenges posed by EM including dealing with poorly defined multifactorial diseases, making assessments without standard assessment tools, interpreting lab investigation without comparative data or established cause-and-effect relationships and a lack of educational resources and defined education pathways. Perhaps the greatest challenge posed by EM is that most chronic diseases may be caused or exacerbated by environmental exposures, and thus, EM is relevant to all fields of medicine [1]. There is therefore a need to inform all clinicians about toxicant exposures and adverse health effects and educate them about the factors that influence individual susceptibility to toxicant exposures. This may be achieved through the development of standardised environmental exposure surveys and other tools to assess, monitor and map exposures and their health effects. It will also require the inclusion of EM education in standard medical curricula and post-graduate training and the publishing of information about EM in general medical textbooks and medical journals, which is a need that has been voiced by numerous organisations and researchers over many decades [29–37].

### Limitations

Whilst this paper has identified clear themes, our research is limited by inherent limitations of qualitative research which include the biases we bring as clinician researchers who decide on the questions to ask and how responses are interpreted. The study population was also limited to Australia and New Zealand, and the finding of two distinct groups clearly arose from the organisations from which participants were recruited. Our findings therefore may not be representative of EM clinicians elsewhere, and we cannot be sure that the inclusion of additional participants from other sources would not have led to additional or different themes emerging.

EM is certainly a complex field that requires an integration of top-down and bottom-up approaches in order to understand potential sources of toxicant exposures and their health impacts, monitor and mitigate individual exposure profiles and risk factors and evaluate measures to minimise exposures and their effects. These tasks are not only relevant to all clinicians, they also require engagement from the wider community, including policy makers and individual citizens, and the tools to achieve this are becoming widely available to the community. There are now multiple community-led, citizen-science campaigns and ‘crowd-and-the-cloud’ projects enabling the general community to participate in scientific projects that monitor exposures to toxicants at a personal level [38–40], collect data on the occurrence of disease geographically [41], identify the sources and impact of different exposures and assess the efficacy of public health campaigns and individual treatment protocols and expand environmental health literacy [42]. Such efforts will be greatly assisted through the development of standardised exposure tools that combine medical and exposure history data with biomarker information along with data from the growing number of environmental sensors. This has implications for all future clinicians, educators and patients and will change the way that EM is practiced in the future.

## Conclusions

EM is relevant to all fields of medicine yet is currently divided between integrative medical practitioners using patient-centred approaches in private-practice settings and occupational and environmental physicians using public-health approaches in workplace settings. Clinicians practicing EM face challenges in assessing toxicant loads in the absence of comprehensive educational resources, definitive laboratory tests, established dose-response relationships or exposure history tools. Whilst there is widespread agreement that an exposure history is the most useful clinical tool for assessing toxicant exposures, there are no standardised assessment tools, and the development of such tools has the potential to significantly advance the clinical practice of EM and expand its reach across clinical disciplines. Engaging patients and the wider community has further potential to integrate knowledge about toxicant exposures, individual susceptibility, lifestyle and work choices and usher in a new era of personalised medicine that accounts for the impact of the environment on the health of populations and individuals.

## Abbreviations

ACNEM: Australasian College of Nutritional and Environmental Medicine
AFOEM: Australasian Faculty of Occupational and Environmental Medicine
EM: Environmental medicine
IPs: Integrative medical practitioners
OEPs: Occupational and environmental physicians
